# High-sensitivity flip chip blue Mini-LEDs miniaturized optical instrument for non-invasive glucose detection

**DOI:** 10.1186/s11671-023-03948-9

**Published:** 2024-01-04

**Authors:** Zhi Ting Ye, Shen Fu Tseng, Shang Xuan Tsou, Chun Wei Tsai

**Affiliations:** 1 Department of Mechanical Engineering, Advanced Institute of Manufacturing with High-Tech Innovations, National Chung Cheng University, 168, University Rd., Min-Hsiung, Chia-Yi, 62102 Taiwan, ROC; 2 Graduate Institute of Photonics and Optoelectronics, National Taiwan University, No. 1, Sec. 4, Roosevelt Rd., Taipei, 106319 Taiwan, ROC

## Abstract

The colorimetric detection of glucose typically involves a peroxidase reaction producing a color, which is then recorded and analyzed. However, enzyme detection has difficulties with purification and storage. In addition, replacing enzyme detection with chemical methods involves time-consuming steps such as centrifugation and purification and the optical instruments used for colorimetric detection are often bulky and not portable. In this study, ammonium metavanadate and sulfuric acid were used to prepare the detection solution instead of peroxidase to produce color. We also analyzed the effect of different concentrations of detection solution on absorbance sensitivity. Finally, a flip chip blue Mini-LEDs miniaturized optical instrument (FC blue Mini-LEDs MOI) was designed for glucose detection using optics fiber, collimating lenses, a miniaturized spectrometer, and an FC Blue Mini-LEDs with a center wavelength of 459 nm. While detecting glucose solutions in the concentration range of 0.1–10 mM by the developed MOI, the regression equation of y = 0.0941x + 0.1341, R^2^ of 0.9744, the limit of detection was 2.15 mM, and the limit of quantification was 7.163 mM. Furthermore, the preparation of the detection solution only takes 10 min, and the absorbance sensitivity of the optimized detection solution could be increased by 2.3 times. The detection solution remained stable with only a 0.6% decrease in absorbance compared to the original after storing it in a refrigerated environment at 3 °C for 14 days. The method proposed in this study for detecting glucose using FC blue light Mini-LEDs MOI reduces the use of peroxidase. In addition, it has a wide detection range that includes blood as well as non-invasive saliva and tear fluids, providing patients with a miniaturized, highly sensitive, and quantifiable glucose detection system.

## Introduction

Glucose is the primary energy source for the metabolism of most cells in the body and is crucial for the brain [1]. In terms of energy utilization, the adult brain requires more than 20% of the body's total energy [2]. Almost all the brain's energy is generated from glucose; thus, it consumes approximately 60% of the body's glucose (about 120 g per day) [3]. The blood glucose concentration in healthy individuals is typically between 4.9 and 6.9 mM before and after meals [4]. Excessive glucose can damage cells and organ systems [5]. Diabetes is a chronic disease characterized by abnormally high blood glucose levels due to impaired insulin action or secretion [6]. The two most common forms of diabetes are type 1 diabetes, which is characterized by a decrease in insulin production, and type 2 diabetes, which is characterized by an impaired insulin response. These pathophysiological conditions can lead to chronic hyperglycemia, resulting in many metabolic abnormalities [7, 8]. According to recent medical research, the global prevalence and incidence of diabetes have increased rapidly in the last decade [9]. Over 80% of diabetes patients live in developing countries. Aging, urbanization, and lifestyle changes are the main determinants underlying their rapid increase [10]. Due to urban lifestyle, reduced physical activity leading to obesity is the main factor driving the increasing diabetes incidences [10–12]. High blood glucose damages small and large blood vessels, increasing the risk of microvascular and macrovascular complications [13]. In addition, medical research showed that people with diabetes have a significantly higher risk of cardiovascular disease than other individuals [14]. Dietary habits significantly impact diabetes, with high-calorie Western diets and sedentary lifestyles being the leading causes of the disease [15]. Furthermore, excessive consumption of refined grains or sugary drinks can also increase the risk of obesity and diabetes [16–18]. Therefore, humans must manage their glucose levels to reduce the risk of complications and disease progression [19]. In trials, glucose management has been shown to benefit microvascular complications such as retinopathy, nephropathy, and neuropathy [20].

Portable blood glucose monitors connected to disposable test strips have been developed for analyzing blood glucose levels by collecting blood on the test strip using a needle [21]. However, the inconvenience and risk of infection associated with invasive testing are primary concerns for most patients; therefore, a non-invasive alternative method is required to monitor blood glucose levels [22]. According to medical statistics, the glucose concentrations in the sweat, saliva, and tears of healthy individuals are between 0.06–0.11 mM, 0.23–0.38 mM, and 0.05–0.5 mM, respectively [23]. With the advancements of computer, communications, consumer-electronics (3C) technology, wearable biosensors can be used to monitor glucose concentration in sweat as an alternative to blood testing [24, 25]. However, wearable devices cannot perfectly follow the micro-curvature of the skin due to wrinkles, which limits signal accuracy [26]. During the enzymatic reaction of converting glucose into its hydrogen peroxide through the colorimetric method, the change in glucose concentration can be quickly measured to determine if there is abnormal blood sugar [27, 28]. Compared to the prior studies, the main method of detecting glucose is based on the chemical reactions of H_2_O_2_. In our studies, we provide an innovation method of detecting glucose via the development of detection solution and flip chip blue Mini-LEDs. The purpose of this glucose detection is to reduce the use of peroxidase enzymes. In addition, with the development of smartphone-based applications, the color change results caused by the colorimetric method can be captured by taking a photo. The photo can be analyzed in real-time to evaluate the disease index of the sample [29–31]. The principle of image analysis is to convert the photo into R, G, and B values through image processing software and analyze the degree of color change caused by the increase in glucose concentration to achieve glucose index detection [32, 33]. In regression analysis, the coefficient of determination (R^2^) is used to predict the correlation with the actual situation. When R^2^ = 0.8, it indicates that the performance of the regression line is very good [34]. Some optical detection systems based on quantum dots and smartphones are proposed to detect glucose [35, 36]. Besides, the smartphone base sensors can also use ambient light to detect glucose [37]. However, the smartphone resolution may affect the analysis results [38]. Spectroscopy is another method for glucose detection [39, 40]. The optical sensing chip is a way for detecting glucose [41]. Moreover, nano enzyme is also developed to detect glucose by a spectrophotometer [42, 43]. Subtracting the peroxidase reaction is the main problem to be solved by the above methods. Because enzyme detection has difficulties in purification, easy deterioration, and difficult storage [44]. In addition, the high weight and large size of traditional detection devices make them unsuitable for real-time detection [45]. Mini LEDs have the advantages of small size, high color purity, power saving, and high efficiency. When used in optical detection instruments, they can significantly reduce the size and improve convenience y[46, 47].

To solve the problem of using peroxidase in glucose detection, the long preparation time for the detection solution, and to reduce the size of the detection equipment, we used ammonium metavanadate and sulfuric acid to prepare the detection solution, analyzed the sensitivity of absorbance to different concentrations of the detection solution, the preparation of the detection solution only takes 10 min, and the absorbance sensitivity of the optimized detection solution could be increased by 2.3 times. Finally, developed the FC Blue Mini-LEDs MOI for detecting glucose solutions in the concentration range of 0.1–10 mM, the regression equation of y = 0.0941x + 0.1341, R^2^ of 0.9744, the LOD was 2.15 mM, and the LOQ was 7.163 mM. The current study achieves a highly-sensitive, fast, low-cost, and portable method for glucose quantification. In this study, we focus on developing the detection method for non-invasive glucose with Mini-LEDs miniaturized optical instrument. The architecture we proposed is to detect the glucose in tears or saliva, not blood sugar directly. Besides, the glucose will be present in any body fluid in our body. Hence, this experiment is based on the concentration of glucose in tears and saliva. That is why we mentioned that we can execute the non-invasive glucose detection via high-sensitivity flip chip blue Mini-LEDs miniaturized optical instrument. Regarding the real sample experiments and data demonstration, these are our next research work.

## Materials and methods

### Chemicals and materials

D-( +)-glucose (CAS: 50-99-7) and glucose oxidase (CAS: 9001-37-0, enzyme activity > 100 units/mg) were purchased from Sigma-Aldrich (Sigma-Aldrich, Inc, USA). Ammonium metavanadate (CAS: 7803-55-6) was purchased from Scharlau (Scharlau, Inc, Spain). Hydrogen peroxide (H_2_O_2_, 35%, CAS: 7722-84-1) was purchased from Shimakyu Chemical (Shimakyu Chemical, CO., LTD., Japan). Sulfuric acid (H_2_SO_4_, CAS: 7664-93-3) was purchased from Kojima Chemical (Kojima Chemical, CO., LTD., Japan). In this experiment, distilled water was used to prepare all chemical solutions. The absorbance of the solutions was measured using a Hitachi U-3900 spectrophotometer (Hitachi, Ltd., Tokyo, Japan) with a 1 cm quartz cell to measure the changes in absorbance.

### Detection principle

The glucose detection method comprises two chemical reactions, Eq. (1) and Eq. (2). Firstly, glucose oxidase catalyzes glucose into hydrogen peroxide and gluconic acid. Then, ammonium metavanadate and sulfuric acid are added to react with hydrogen peroxide, ammonium metavanadate, and sulfuric acid, producing peroxovanadium complex (NH_4_[VO(O_2_)SO_4_]). Since the absorbance peak of the peroxovanadium complex is at 452 nm, the glucose content can be calculated by detecting the absorbance of the solution at 452 nm.

1 $${\text{ O}}_{2} + {\text{glucose}}\mathop{\longrightarrow}\limits^{{{\text{Glucose Oxidase }}}} {\text{H}}_{2} {\text{O}}_{2} + {\text{Gluconic}}\;{\text{ Acid }}$$

2 $${\text{H}}_{2} {\text{O}}_{2} + {\text{NH}}_{4} {\text{VO}}_{3} + {\text{H}}_{2} {\text{SO}}_{4} \to {\text{NH}}_{4} \left[ {{\text{VO}}({\text{O}}_{2} } \right){\text{SO}}_{4} ] + {\text{H}}_{2} {\text{O}}$$

### Preparation and optimization of detection solution

0.581 g of ammonium metavanadate powder was added to 50 mL of sulfuric acid (0.5 M). The solutions were stirred with a magnetic stirrer at 500 rpm for 10 min until they were uniformly mixed to form a 0.1 M ammonium metavanadate sulfuric acid solution. Then, 0.5 M sulfuric acid was added to dilute the solution into six different concentrations of detection solutions: 0.01, 0.02, 0.04, 0.06, 0.08, and 0.1 M. Finally, hydrogen peroxide and the detection solution were mixed in 2:1 ratio to form a 3 mL solution for detection. According to the Beer-Lambert law, when the absorbance is 2, 99% of the light is absorbed. We choose the concentrations with an absorbance of less than 2 while optimizing the sensitivity of the detection solution to ensure that the final detection solution does not absorb too much light, which could affect the accuracy of the microspectrophotometer. Since hydrogen peroxide is close to the highest glucose concentration to be analyzed at 10 mM, when diluted 1000 times, it will be used as the sample for analyzing ammonium metavanadate sulfuric acid detection solution.

### Design a flip chip blue Mini-LEDs MOI for glucose detection

**Fig. 1:**
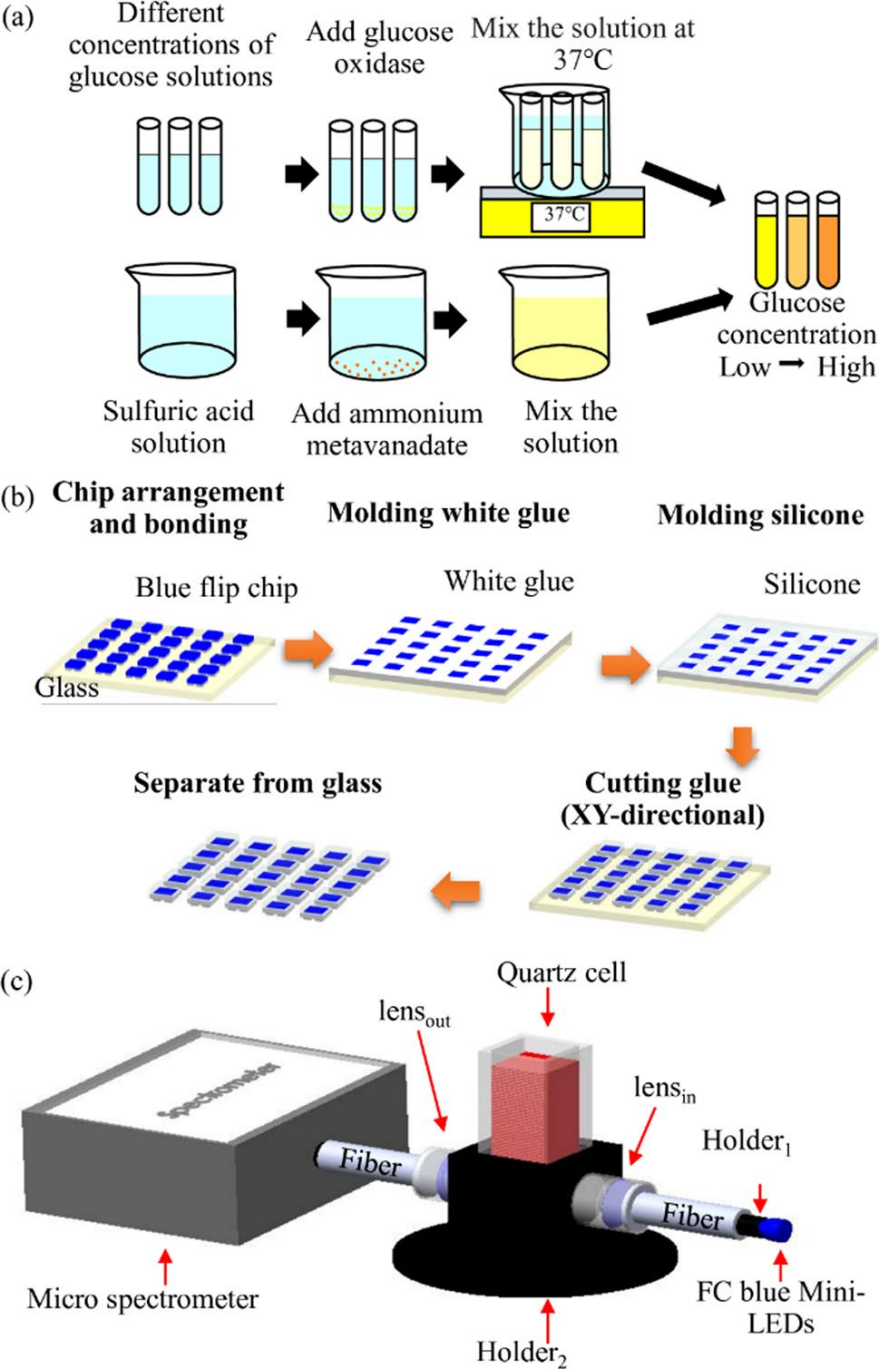
Proposed miniaturized optical glucose detection instrument. **a** The preparation process of glucose detection solution. **b** The fabrication process of the Flip chip blue Mini-LEDs. **c** Schematic diagram of miniaturized optical glucose detection instrument

Figure 1 shows the flip chip blue Mini-LEDs MOI for glucose detection. The preparation process of the glucose detection solution is shown in Fig. 1a. First, 0.09 g of glucose was added to 10 mL of water to dissolve completely. Then, water was added to dilute the solution to 50 mL. The solutions were stirred at 500 rpm with a magnetic stirrer for 10 min until they were uniformly mixed to obtain the desired 10 mM glucose solution. The glucose solution was further diluted into seven different concentrations: 0.1, 0.4, 0.8, 1, 4, 8, and 10 mM, and catalyzed by 5 mg/mL of glucose oxidase. The beaker containing solution was heated with a hot plate and kept at 37 °C to simulate human body temperature, which was monitored by TES-1380 (TES Electrical Electronic Corp., Taipei, Taiwan). After completing catalysis, the detection solution was added dropwise to complete the reaction. The ratio of adding glucose, glucose oxidase, and detection solution was 9:1:5. The FC blue Mini-LEDs used in this study were produced through chip arrangement and bonding, molding white glue and silicone, cutting glue, and separate from glass approaches, as shown in Fig. 1b. The chip has a length, width, and height of 101.6 μm, 152.4 μm, and 150 μm, respectively. The package size has a length, width, and height of 800 μm, 800 μm, and 150 μm, respectively. The schematic diagram of the miniaturized optical instrument for glucose detection is shown in Fig. 1c. Flip chip (FC) blue Mini-LEDs were used as the light source, combined with two optical fibers with a length, core diameter, and numerical aperture of 30 cm, 600 μm, and 0.22, respectively. The collimating lens with a back focal length and numerical aperture of 10 mm and 0.22, respectively, and a micro-spectrometer SE1030-025-FUVN (OtO Photonics, Inc., Hsinchu City, Taiwan) to design the miniaturized optical instrument. Finally, the computer analyzed the detection results of different glucose concentrations. A black sleeve was used as Holder_1_ to fix the FC blue Mini-LEDs and optical fibers for adjusting the light source and preventing light leakage, while the position of the quartz groove was fixed by Holder_2_.

### Experimental procedure

The glucose detection experiments using the MOI developed in this study were conducted in a dark room. First, six different concentrations (0.01–0.1 M) of ammonium metavanadate sulfuric acid detection solution were prepared, and the optimal detection liquid concentration was confirmed by diluting hydrogen peroxide 1000 times. After preparing the detection liquid, 5 mg/mL of glucose oxidase and seven different concentrations (0.1–10 mM) of glucose were catalyzed and reacted at 37 °C. To detect the glucose concentrations, HITACHI U-3900 was used to measure the results at different concentrations. Next, a micro-optical instrument was designed using FC blue Mini-LEDs as the light source, combined with fiber optics, collimating lenses, and a micro-spectrometer to measure and analyze the glucose concentrations. Finally, the glucose concentration measurement results obtained using the HITACHI U-3900 and the FC blue Mini-LEDs MOI were compared.

## Results and discussion

### Optimization of the detection solution

**Fig. 2:**
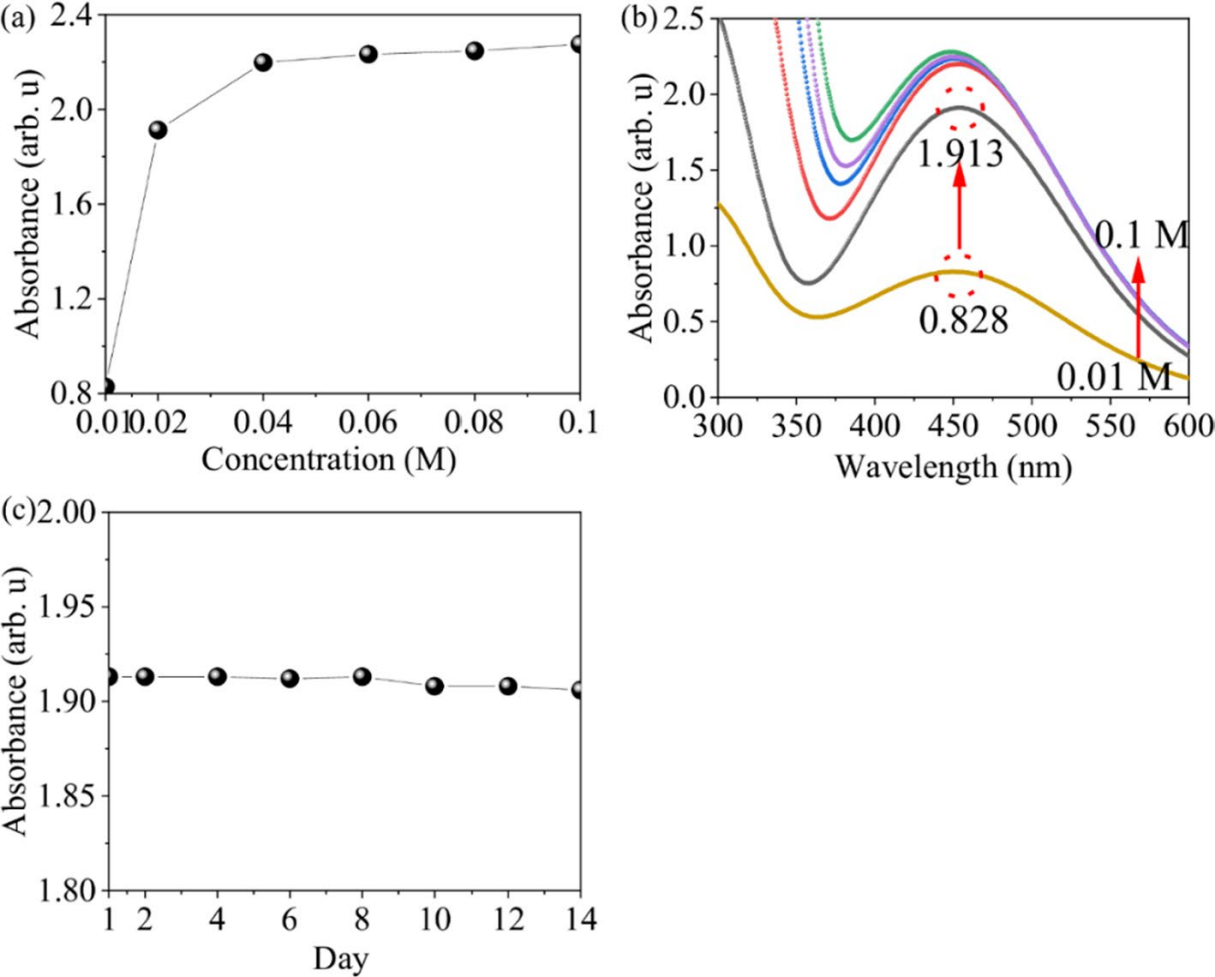
The optimization of the detection solution **a** color change after chemical reaction and absorbance change at 452 nm. **b** Absorbance spectra of different concentrations of detection solutions. **c** Changes in the absorbance of the detection solution after storing in a refrigerated environment for 14 days

The Optimization of the detection solution result as shown in Fig. 2. The color change of the reaction results of adding 1000 times diluted hydrogen peroxide to six different concentrations of detection solution (0.01–0.1 M) is shown in Fig. 2a, where the solution gradually changes from orange-red to deep red as the concentration of detection solution increases from left to right, indicating an increase in the number of peroxovanadium complexes, and the measurement values of the absorbance peak of peroxovanadium complexes at 452 nm, which tends to saturate when the concentration of the detection solution reaches 0.04 M. Figure 2b represents the absorbance spectra of the detection solution, and the absorption peak can be observed at 452 nmA peroxide complex is formed and has an absorption peak at 452 nm during the reaction between detection solution and hydrogen peroxide. In addition, the absorbance at 452 nm increases and the absorbance spectra shows a blue shift with the increasement of the concentrations of detection solution. The measurement results indicate that the absorbance tends to saturate when the concentration reaches 0.04 M and a higher absorbance above 2. We speculate that the reason for the blue shift in the absorption spectrum is that when the concentration of the detection solution exceeds 2, only 1% of the light can penetrate. The measurement error range of the absorption values in this analytical instrument is below 2%. Therefore, a slight shift in the absorption spectrum occurs. As a result, the analysis in this paper focuses on detection solutions with absorption values all below 1.2. Therefore, considering the stability and practicality of the detection solution, a concentration of 0.02 M was chosen for this experiment. The absorbance of the detection solution increased from 0.828 arb. u at 0.01 M to 1.913 arb. u at 0.02 M, resulting in a 2.3-fold increase in absorbance sensitivity. The absorbance measurements at 452 nm of the detection solution stored at 3 °C for 14 days are shown in Fig. 2c. After 14 days of storage, the absorbance decreased by 0.6% compared to the original, verifying the excellent storage stability of the detection solution developed in our study.

### Glucose detection by Hitachi U-3900

**Fig. 3:**
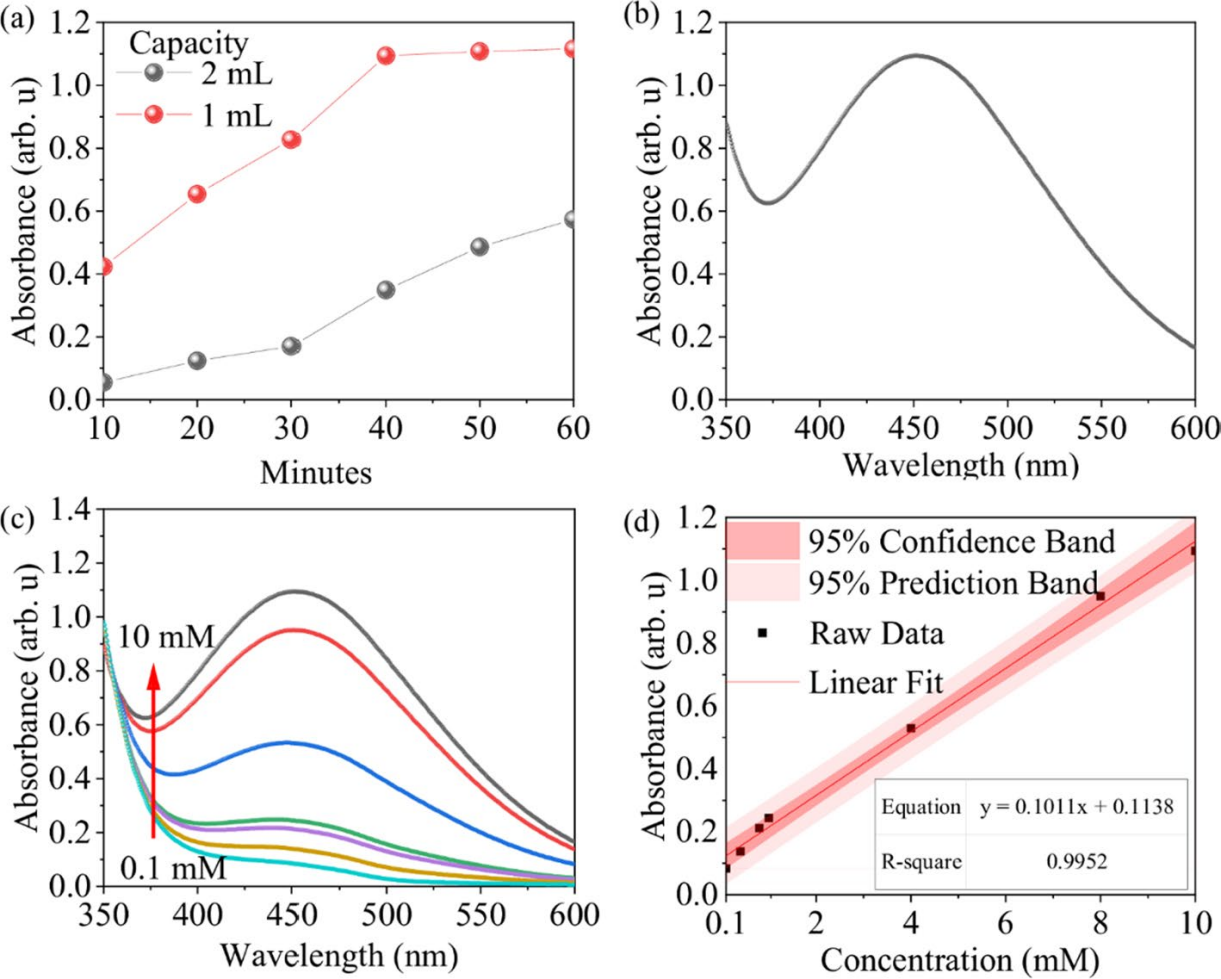
Hitachi U-3900 detects 7 different concentrations of glucose. **a** Catalysis time analysis for two different volumes. **b** Absorbance spectrum using 1 mL catalysis. **c** Absorbance spectrum measurement. **d** Linear analysis

Firstly, 5 mg/mL of glucose oxidase was used to catalyze 10 mM of glucose at 37 °C. The temperature of the heating plate was set to 40 °C so that the final water temperature could be maintained at 37 ± 0.5 °C. The absorbance results are presented in Fig. 3. When the glucose-to-glucose oxidase ratio was 9:1, solutions of 1 mL and 2 mL were prepared for detection, and their reaction results are shown in Fig. 3a. Usually, the water temperature will rise more slowly when the aqueous solution volume is higher. The reaction result shows the absorbance of solution volume 2 mL did not reach the reaction saturation within the experimental time, but the absorbance of solution volume 1 mL has reached the reaction saturation. Hence, the absorbance of solution volume 1 mL is higher than solution volume 2 mL. When the solution volume was 2 mL (black line), the absorbance did not reach saturation even after 60 min of catalysis. In contrast, when the solution volume was 1 mL (red line), the absorbance tended to saturate after 40 min of catalysis. This was because the increase in the solution’s volume might slow the temperature rise and thus reduce the catalytic rate. Therefore, the glucose and glucose oxidase volume used in subsequent experiments was kept at 1 mL. After 40 min of catalysis of 1 mL of glucose and glucose oxidase, the absorbance spectrum is shown in Fig. 3b. Since the generation of the peroxovanadate complex has an absorbance peak at 452 nm, the increase in the absorbance spectrum below 400 nm could be due to the intrinsic absorbance of the detection solution. Using 5 mg/mL of glucose oxidase, 0.1–10 mM glucose solutions at seven different concentrations were catalyzed at 37 °C, and the results were detected using a 0.02 M detection solution, as shown in Fig. 3c. Generally, ordinary people have blood sugar as 5.6 mM, sweat sugar as 0.11 mM, and tear glucose as 0.5 mM. Compared to ordinary people, the patients with hyperglycemia have higher blood sugar, sweat sugar and tear glucose. Hence, the author takes an integer to establish a regression line between 0.1 mW to 10 mW in order to cover the above range. As the concentration of the glucose solution increases from right to left, the color changes gradually from light yellow to orange-red and finally to dark red, the absorbance spectra. As the amount of the peroxovanadate complex increases, the peak absorbance at 452 nm also increases. Moreover, the lower absorbance at 350 nm for 8 mM and 10 mM concentrations is due to a decrease in the detection solution concentration caused by the chemical reaction of hydrogen peroxide with the detection solution. The regression equation for detecting 0.1–10 mM glucose solutions at seven different concentrations is y = 0.1011x + 0.1138, with an R^2^ of 0.9952, a LOD (3σ/s) of 0.92 mM, and a LOQ (10σ/s) of 3.08 mM, as shown in Fig. 3d. Besides, The prediction 95% confidence interval (the interval with error less than 5) estimated based on the software formula is y = (0.1011 ± 0.10182)x + (0.1138 ± 0.5189). The 95% confidence interval of the actual measurement (the interval with an error less than 5) is y = (0.1011 ± 0.0032)x + (0.1138 ± 0.0161).

### Glucose detection using flip-chip blue Mini-LEDs MOI

**Fig. 4:**
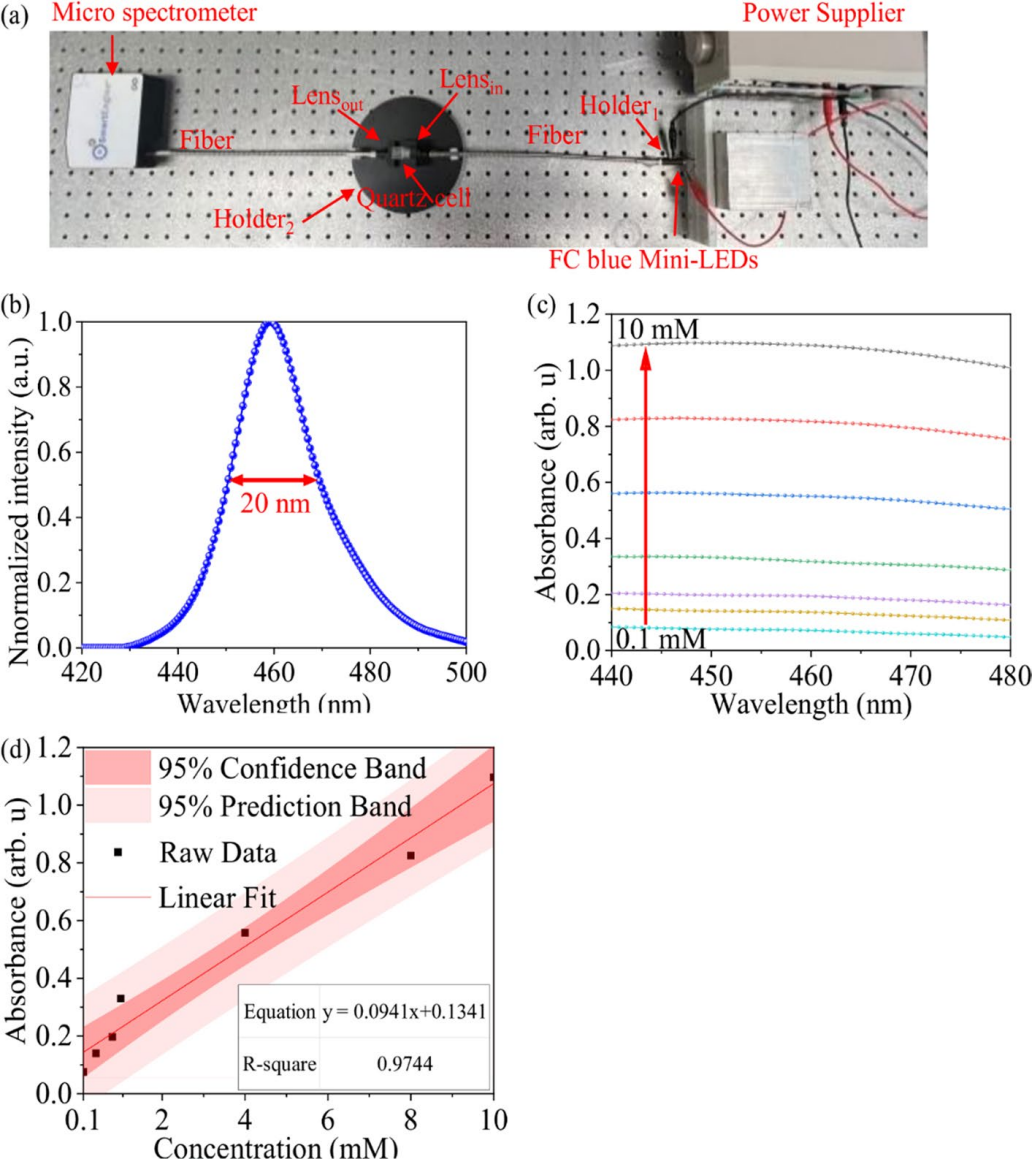
FC blue Mini-LEDs MOI detection of glucose. **a** Entity diagram of the FC Blue Mini-LEDs MOI. **b** Normalized spectrum. **c** Absorbance spectrum. **d** Linear analysis

The FC Blue Mini-LEDs MOI designed for glucose detection in this study is shown in Fig. 4. Figure 4a represents the optical setup, which includes FC Blue Mini-LEDs, fiber, the lens in, lens out, micro spectrometer, and Holder_1_ and Holder_2_, which were used to fix the LEDs and quartz cell. Figure 4b shows the normalized spectrum of the FC Blue Mini-LEDs, with a center wavelength of 459 nm and a full width at half maximum (FWHM) of 20 nm. When using the proposed FC Blue Mini-LEDs MOI to detect glucose, it is essential to ensure that the intensity of the light source does not exceed the detection limit of the spectrometer. The FC blue Mini-LEDs were driven by a voltage of 2.55 V and a current of 20 mA, and the integration time of the spectrometer was fixed at 850 μs. The detection results of seven different glucose concentrations ranging from 0.1 to 10 mM are provided in Fig. 4c. The absorbance spectrum, which only displays the detection results at wavelengths between 440 and 500 nm due to the use of monochromatic Mini-LEDs, and the absorbance peak is located at 452 nm. The regression equation of the detection results for the seven different glucose concentrations ranging from 0.1 to 10 mM is y = 0.0941x + 0.1341, with an R^2^ of 0.9744, LOD of 2.15 mM, and LOQ of 7.16 mM, as shown in Fig. 4d.

### Comparing detection linearity between FC Blue Mini-LEDs MOI and Hitachi U-3900

Table 1. shows the comparison of glucose detection results using Hitachi U-3900 and the proposed FC Blue Mini-LEDs MOI. The Hitachi U-3900 and FC Blue Mini-LEDs MOI were used to measure glucose solutions with concentrations ranging from 0.1 to 10 mM at 452 nm. The regression equations and R^2^ values were y = 0.1011x + 0.1138, R^2^ = 0.9952, and y = 0.0941x + 0.1341, R^2^ = 0.9744, respectively. Compared to Hitachi U-3900, the proposed FC Blue Mini-LEDs MOI has more advantages, such as compact, flexible, easy to install, and can be carried around. Hence, the comparison suggested that the FC Blue Mini-LEDs MOI proposed in this study could be efficiently used as a reference method for glucose detection.

**Table 1:** Comparing Detection Linearity between FC Blue Mini-LEDs MOI and Hitachi U-3900

Method	Correlation of determination (R^2^)	Linear equation
HITACHI U-3900	0.9952	y = 0.1011x + 0.1138
FC Blue Mini-LEDs MOI	0.9744	y = 0.0941x + 0.1341

## Conclusion

In this study, the glucose detection method was developed using ammonium metavanadate and sulfuric acid to replace the enzyme-based detection method, the preparation of the detection solution only takes 10 min which solves the problem of enzyme purification, long preparation time, and storage difficulties. The developed detection solution was stored at 3 °C for 14 days, and the absorbance intensity decreased only by 0.6%. Additionally, the absorbance sensitivity was improved by 2.3 times by preparing the glucose detection solution. Using Hitachi U-3900 to detect glucose solutions with concentrations ranging from 0.1 to 10 mM, the regression equation was y = 0.1011x + 0.1138, R^2^ = 0.9952, LOD was 0.92 mM, and LOQ was 3.08 mM. Furthermore, to solve the problem of the large size of traditional analytical instruments, this study developed the FC Blue Mini-LEDs MOI with a central wavelength of 459 nm to detect total glucose solutions with concentrations ranging from 0.1 to 10 mM. The regression equation was y = 0.0941x + 0.1341, R^2^ = 0.9744, LOD was 2.15 mM, and LOQ was 7.16 mM. The proposed FC Blue Mini-LEDs MOI method reduces the use of peroxidase enzymes and can detect a wide range of glucose concentrations, including blood, non-invasive saliva, and tears. This method provides patients with a miniaturized, highly sensitive, and quantifiable glucose detection method without requiring complex equipment and offers advantages such as reduced detection time, portability, and a reduction in instrument size and weight.

## Data Availability

The data presented in this study are available on request from the all authors.

## Abbreviations

FC blue Mini-LEDs: Flip chip blue Mini-LEDs
MOI: Miniaturized optical instrument
FeCl3: Ferric chloride
Mini-LEDs: Mini-light-emitting diodes
LOD: Limit of detection
LOQ: Limit of quantification
FWHM: Full width at half maximum
R^2^: Correlation of determination
